# Supplementary material for: Lipid and Protein Oxidation of Brown Rice and Selenium-Rich Brown Rice during Storage
Source: Foods. 2022 Dec 1;11(23):3878. doi: 10.3390/foods11233878 (PMC9737139; doi:10.3390/foods11233878)
Supplement: Supplementary file 1 [file foods-11-03878-s001.zip › foods-2038602-supplementary.pdf]

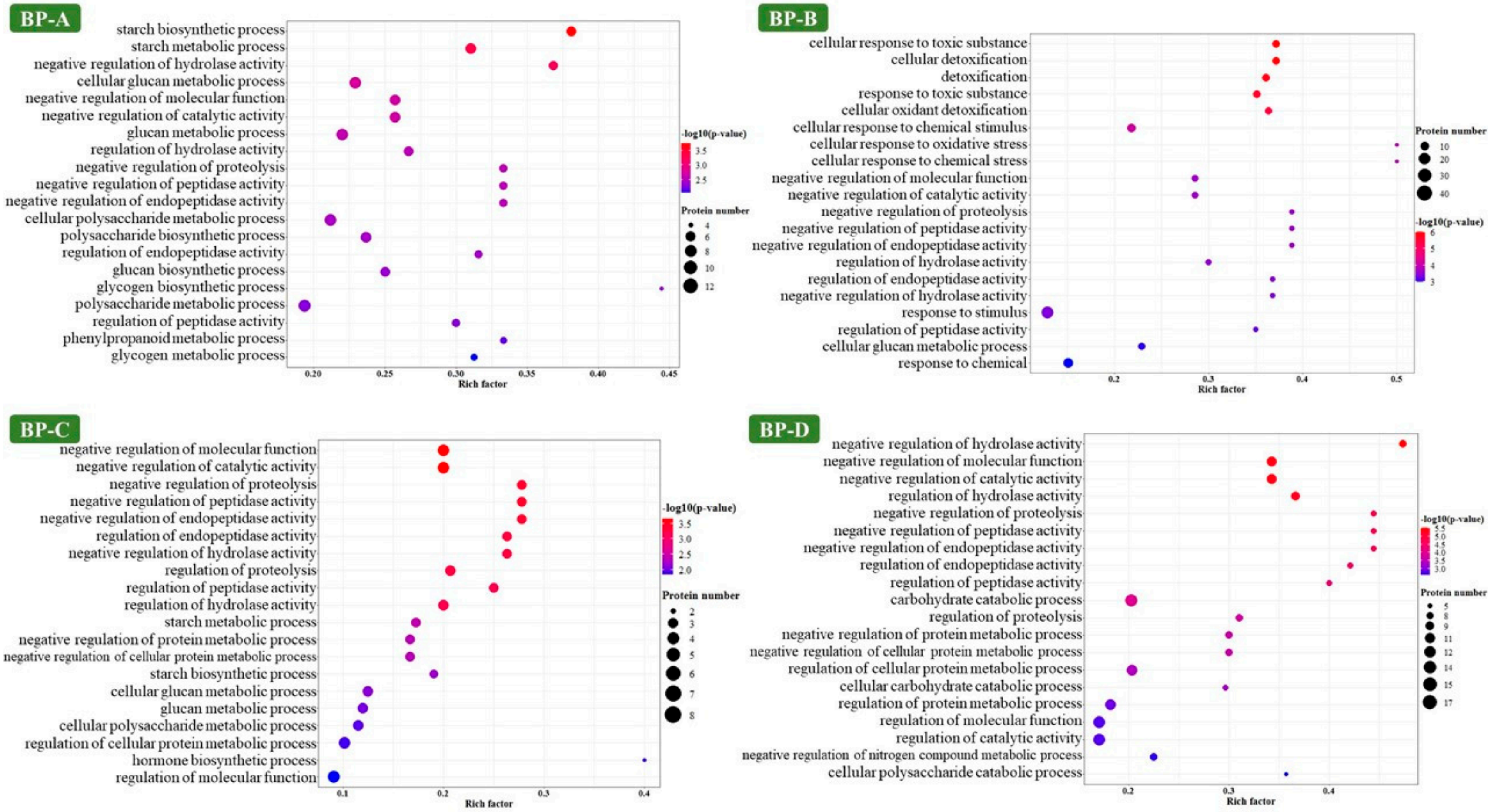
BP-A
starch biosynthetic process
starch metabolic process
negative regulation of hydrolase activity
cellular glucan metabolic process
negative regulation of molecular function
negative regulation of catalytic activity
glucan metabolic process
regulation of hydrolase activity
negative regulation of proteolysis
negative regulation of peptidase activity
negative regulation of endopeptidase activity
cellular polysaccharide metabolic process
polysaccharide biosynthetic process
regulation of endopeptidase activity
glucan biosynthetic process
glycogen biosynthetic process
polysaccharide metabolic process
regulation of peptidase activity
phenylpropanoid metabolic process
glycogen metabolic process
Rich factor
-log10(p-value)
Protein number
BP-B
cellular response to toxic substance
cellular detoxification
detoxification
response to toxic substance
cellular oxidant detoxification
cellular response to chemical stimulus
cellular response to oxidative stress
cellular response to chemical stress
negative regulation of molecular function
negative regulation of catalytic activity
negative regulation of proteolysis
negative regulation of peptidase activity
negative regulation of endopeptidase activity
regulation of hydrolase activity
regulation of endopeptidase activity
negative regulation of hydrolase activity
response to stimulus
regulation of peptidase activity
cellular glucan metabolic process
response to chemical
Rich factor
Protein number
-log10(p-value)
BP-C
negative regulation of molecular function
negative regulation of catalytic activity
negative regulation of proteolysis
negative regulation of peptidase activity
negative regulation of endopeptidase activity
regulation of endopeptidase activity
negative regulation of hydrolase activity
regulation of proteolysis
regulation of peptidase activity
regulation of hydrolase activity
starch metabolic process
negative regulation of protein metabolic process
negative regulation of cellular protein metabolic process
starch biosynthetic process
cellular glucan metabolic process
glucan metabolic process
cellular polysaccharide metabolic process
regulation of cellular protein metabolic process
hormone biosynthetic process
regulation of molecular function
Rich factor
-log10(p-value)
Protein number
BP-D
negative regulation of hydrolase activity
negative regulation of molecular function
negative regulation of catalytic activity
regulation of hydrolase activity
negative regulation of proteolysis
negative regulation of peptidase activity
negative regulation of endopeptidase activity
regulation of endopeptidase activity
regulation of peptidase activity
carbohydrate catabolic process
regulation of proteolysis
negative regulation of protein metabolic process
negative regulation of cellular protein metabolic process
regulation of cellular protein metabolic process
cellular carbohydrate catabolic process
regulation of protein metabolic process
regulation of molecular function
regulation of catalytic activity
negative regulation of nitrogen compound metabolic process
cellular polysaccharide catabolic process
Rich factor
-log10(p-value)
Protein number

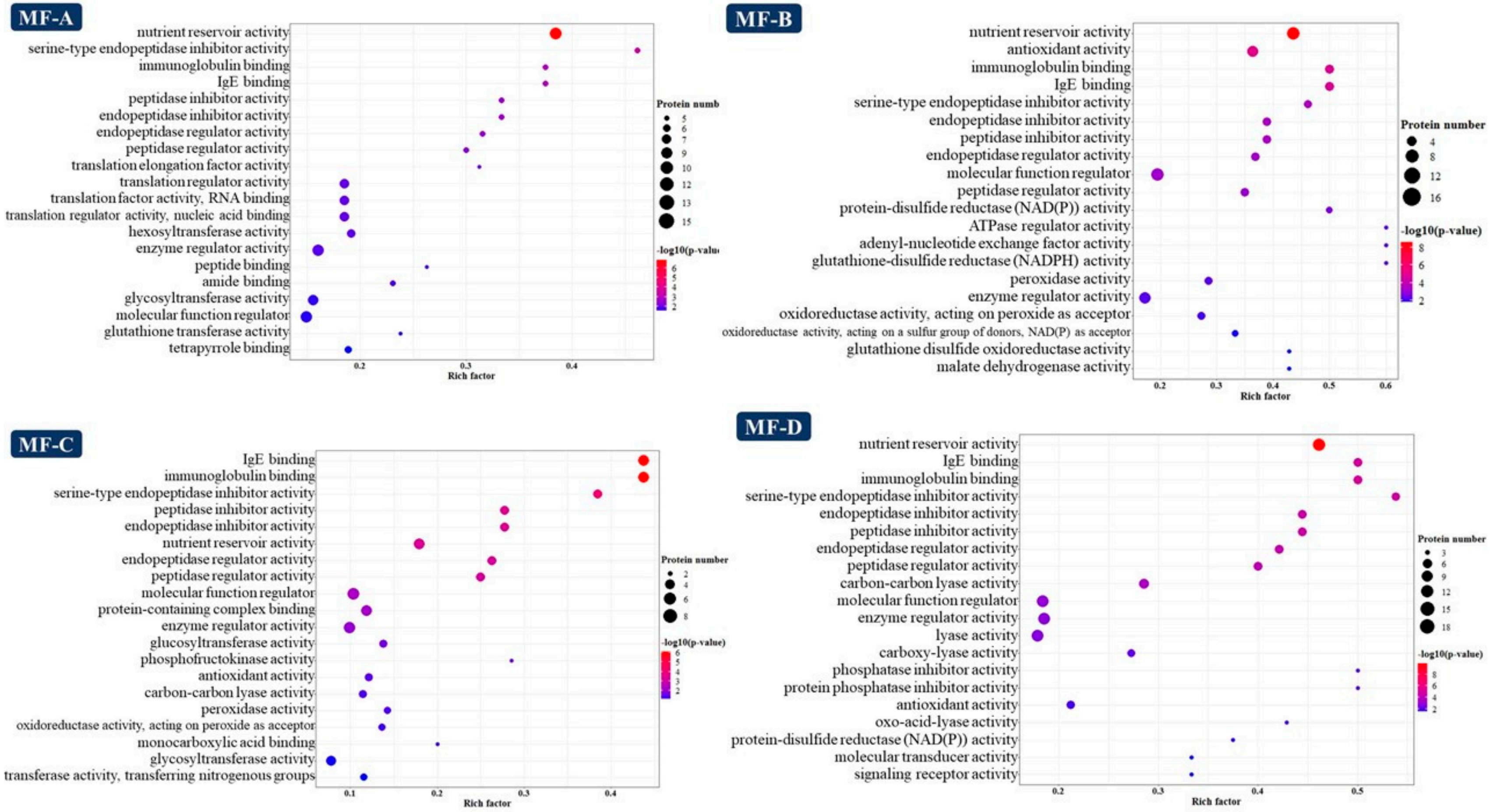

MF-A
nutrient reservoir activity
serine-type endopeptidase inhibitor activity
immunoglobulin binding
IgE binding
peptidase inhibitor activity
endopeptidase inhibitor activity
endopeptidase regulator activity
peptidase regulator activity
translation elongation factor activity
translation regulator activity
translation factor activity, RNA binding
translation regulator activity, nucleic acid binding
hexosyltransferase activity
enzyme regulator activity
peptide binding
amide binding
glycosyltransferase activity
molecular function regulator
glutathione transferase activity
tetrapyrrole binding
Protein numb
-log10(p-valu
Rich factor
MF-B
nutrient reservoir activity
antioxidant activity
immunoglobulin binding
IgE binding
serine-type endopeptidase inhibitor activity
endopeptidase inhibitor activity
peptidase inhibitor activity
endopeptidase regulator activity
molecular function regulator
peptidase regulator activity
protein-disulfide reductase (NAD(P)) activity
ATPase regulator activity
adenyl-nucleotide exchange factor activity
glutathione-disulfide reductase (NADPH) activity
peroxidase activity
enzyme regulator activity
oxidoreductase activity, acting on peroxide as acceptor
oxidoreductase activity, acting on a sulfur group of donors, NAD(P) as acceptor
glutathione disulfide oxidoreductase activity
malate dehydrogenase activity
Protein number
-log10(p-value)
Rich factor
MF-C
IgE binding
immunoglobulin binding
serine-type endopeptidase inhibitor activity
peptidase inhibitor activity
endopeptidase inhibitor activity
nutrient reservoir activity
endopeptidase regulator activity
peptidase regulator activity
molecular function regulator
protein-containing complex binding
enzyme regulator activity
glucosyltransferase activity
phosphofructokinase activity
antioxidant activity
carbon-carbon lyase activity
peroxidase activity
oxidoreductase activity, acting on peroxide as acceptor
monocarboxylic acid binding
glycosyltransferase activity
transferase activity, transferring nitrogenous groups
Protein number
-log10(p-value)
Rich factor
MF-D
nutrient reservoir activity
IgE binding
immunoglobulin binding
serine-type endopeptidase inhibitor activity
endopeptidase inhibitor activity
peptidase inhibitor activity
endopeptidase regulator activity
peptidase regulator activity
carbon-carbon lyase activity
molecular function regulator
enzyme regulator activity
lyase activity
carboxy-lyase activity
phosphatase inhibitor activity
protein phosphatase inhibitor activity
antioxidant activity
oxo-acid-lyase activity
protein-disulfide reductase (NAD(P)) activity
molecular transducer activity
signaling receptor activity
Protein number
-log10(p-value)
Rich factor

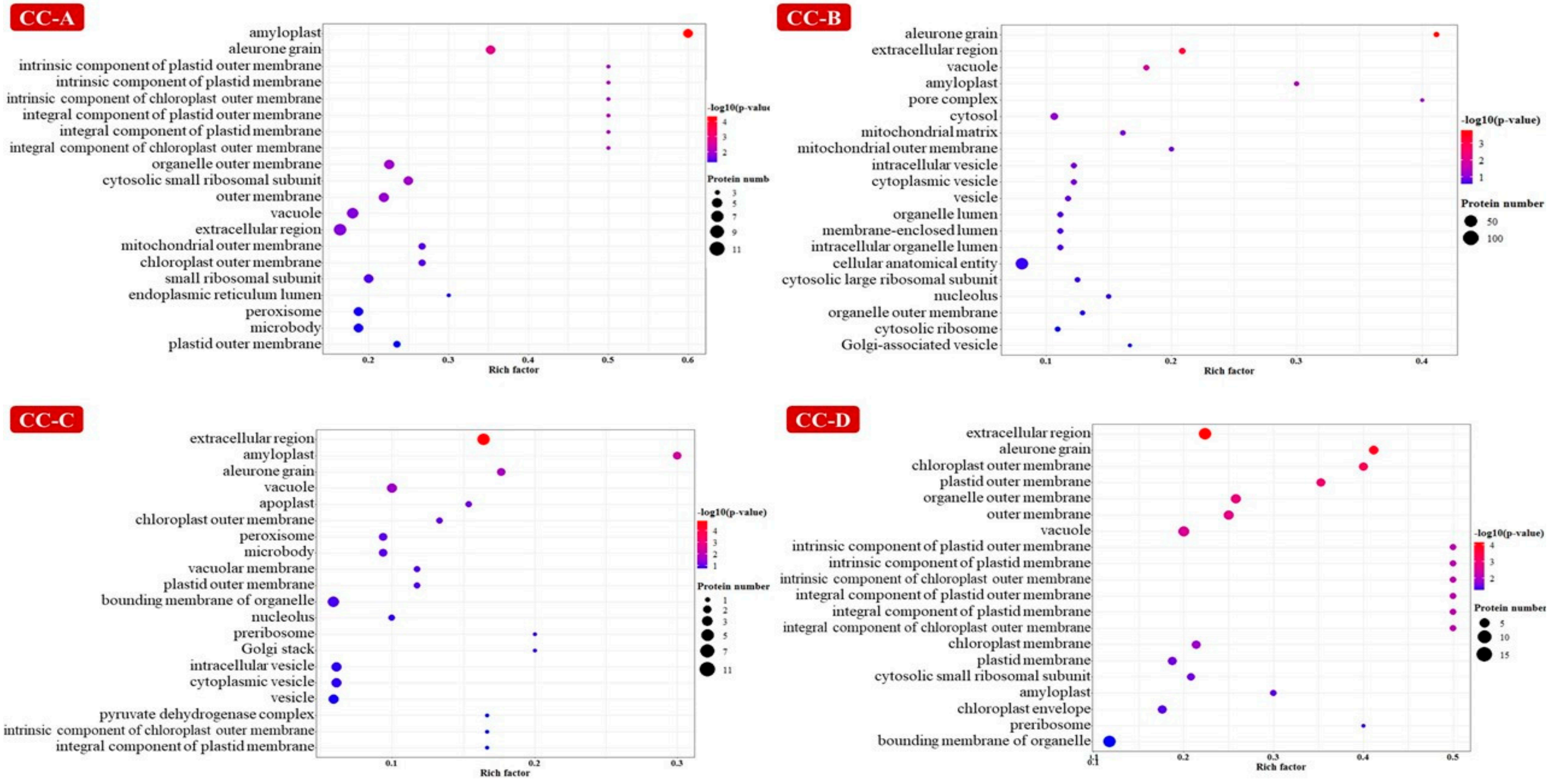

Figure S1. Biological process, cellular component and molecular function of BR and Se-BR. (A refers to BR-0:BR-6; B refers to Se-0:Se-6; C refers to BR-0:Se-0; D refers to BR-6:Se-6). P value is indicated by different colors, redder indicates more significant enrichment; dot size represents the number of DEPs annotated to the item.
